# Carnosine Protects Mouse Podocytes from High Glucose Induced Apoptosis through PI3K/AKT and Nrf2 Pathways

**DOI:** 10.1155/2019/4348973

**Published:** 2019-05-28

**Authors:** Kunxiao Zhao, Ying Li, Ziqiang Wang, Ning Han, Ying Wang

**Affiliations:** ^1^Department of Nephrology, The Third Hospital of Hebei Medical University, Shijiazhuang, Hebei Province 050051, China; ^2^Department of Nephrology, Cangzhou People's Hospital, Cangzhou, Hebei Province 061000, China; ^3^Department of Nephrology, BayanNur Hospital, Bayan Nur, Inner Mongolia Autonomous Region 015000, China

## Abstract

Diabetic nephropathy is the complication of diabetes mellitus that can lead to chronic renal failure. Reactive oxygen species (ROS) production plays an important role in its pathological process. Previous studies showed that carnosine may reduce diabetic nephropathy by antioxidant effect. However, the molecular mechanism of its antioxidant was not fully understood. In the current study, we developed high glucose containing different concentrations of carnosine to reduce ROS levels and podocytes apoptosis, and Cell Counting Kit-8 test was used to observe the cell viability. Carnosine (5-20mM) was found to protect mouse podocytes (MPC5) cells from HG-induced injury. Quantitative real-time PCR, Western blotting, and immunofluorescence staining revealed that high glucose induced ROS levels and podocytes apoptosis were downregulated by PI3K/AKT and Nrf2 signaling pathways. The current findings suggest that carnosine may reduce ROS levels and MPC5 cells apoptosis by PI3K/AKT and Nrf2 signaling pathways activation.

## 1. Introduction

Diabetic Kidney Disease (DKD) is a common complication of both diabetes mellitus type 1 and type 2. Patients with type 2 diabetes have a 40% risk to develop diabetic nephropathy (DN) [1]. ROS production is the significant biochemical change that plays an important role in the development of DN [2]. When oxidative stress occurs, the dynamic balance between oxidative and antioxidative is broken, which leads to the increase of reactive oxygen species and reactive nitrogen production and the decrease of their removal. Proteins, esters and nucleic acids are oxidized and thus cause damage to molecules, cells and collectives. The increase of oxygen free radicals in mitochondria has been proven to be the main cause of diabetic microangiopathy [3]. At the early stages of DN, podocytes apoptosis is the key target for glomerular injury, which precedes the development of DN and deteriorates the kidney function of patients [4, 5].

Carnosine is an endogenous dipeptide. It was first extracted from muscle by Russian chemists Gulewitch and Amiradzibi in 1900 [6]. It was synthesized by *β*-alanine and L-histidine that were catalyzed by ATP-dependent carnosine synthase and hydrolyzed by carnosine synthase. Carnosine has many biological activities, such as buffering physiological acidity and alkalinity, chelating metal ions and antioxidative stress, inhibiting a variety of inflammatory factors, inhibiting advanced glycation end products (AGEs) [7, 8], advancing lipoxidation end products (ALEs) [9], and inhibiting renin-angiotensin-system (RAS) [10] activity.

Nuclear factor erythroid 2-related factor 2 (Nrf2) is thought to play a crucial role in the antioxidant stress system [11]. Recent experimental evidence suggests that phosphatidylinositol-3-kinase/protein kinase B (PI3K/AKT) is involved in the activation of Nrf2 by ROS in response to oxidative stress [12]. Previous studies have shown that the immunoreaction in glomeruli of endothelial nitric oxide synthase in the carnosine + diabetes group decreased compared to diabetic rats, suggesting that revealed carnosine could protect diabetic nephropathy by antioxidant effect [13]. Carnosine prevents apoptosis of glomerular cells and podocyte loss in STZ diabetic rats by reducing the activity of serum carnosinase-1 was reported [14]. PI3K/AKT and Mitogen-activated protein kinases/extracellular signal related kinase (MAPK/ERK) play a significant role in cell apoptosis, survival, and proliferation [15]. Guo et al. [16] demonstrated that carnosine improves diabetic retinopathy via the MAPK/ERK pathway. PI3K/AKT pathway could be potential molecules involved in the protective effect of carnosine on HG-induced apoptosis. Thus, in this study we expected that carnosine could protect mouse podocytes cultured with high glucose from oxidative stress induced apoptosis through PI3K /AKT and Nrf2 pathways.

## 2. Materials and Methods

### 2.1. Chemicals and Reagents

Carnosine (CA), PI3K inhibitor LY294002, and D-glucose were obtained from Sigma (St. Louis, USA). Fetal bovine serum (FBS) was purchased from GIBCO Invitrogen (Carlsbad, CA, USA). RPMI 1640 medium was obtained from Thermo Fisher (Carlsbad, USA). DMEM-F12 medium was purchased from Corning (Steuben County, NY, USA). IFN-*γ* was obtained from MedChem Express (New Jersey, USA). The following antibodies including AKT, Phospho-AKT (473), Phospho-AKT (308), and Cleaved caspase-3 were obtained from Cell Signaling Technology (Beverly, USA). Nephrin was purchased from Abcam (Cambridge, UK). Nrf2, HO-1, Bax, Bcl-2, Histone H3, and *β*-actin were purchased from Proteintech (Chicago, USA).

### 2.2. Cell Culture

Mouse podocytes (MPC5) were cultured in RPMI 1640 medium that contained 10% FBS, penicillin (100 U/ml), streptomycin (100 g/ml), and IFN-*γ* (50 U/ml), at 33°C in growth permissive conditions. To induce differentiation, cells were cultured at 37°C in 95% air/5% CO_2_ without IFN-*γ* for 2 weeks and were used for experiment. The podocytes were cultured in DMEM-F12 (5:1) medium containing normal glucose (NG, 5.5 mmol/L) or high glucose (HG, 30 mM) in the absence or presence of CA (5, 10, 20, 30mM) for 48h. PI3K inhibitor LY294002 (20 *μ*M) was preincubated for 2h.

### 2.3. Cell Viability

Cell viability was detected with a CCK8 kit (MedChem Express, New Jersey, USA). Podocytes were seeded in 96-well plates at a density of 10^3^ -10^5^cells/well and grown in 100 *μ*l complete medium overnight at 37°C with 5% CO_2_. After 24-h treatment, the medium was refreshed and 10ul of the CCK-8 solution was added to each well of the plate. After 2 h of incubation, absorbance was measured at 450 nm using a microplate reader (Bio–Rad Laboratories, Hercules, CA, USA).

### 2.4. Quantitative Real-Time PCR (qRT-PCR)

Total RNA was prepared from cultured podocytes by using the Trizol reagent (Thermo Fisher, Carlsbad, USA) according to the manufacturer's instructions. And 2 *μ*g of total RNA was used to synthesize cDNA with a SuperScript Reverse Transcription Kit (Thermo Fisher, Carlsbad, USA). Quantitative RT-PCR was conducted on Agilent Mx3000P QPCR Systems (Biosystems, USA) with SYBR Green Master Mix (Vazyme, Nanjing, China). Specific primers for Nrf2, Ho-1, and *β*-actin were designed and synthesized by Sangon Biotech (Shanghai, China). Primer sequences were as follows:* Nrf2*: CAGCCATGACTGATTTAAGCAG and CAGCTGCTTGTTTTCGGTATTA.* HO-1*: TCCTTGTACCATATCTACACGG and GAGACGCTTTACATAGTGCTGT.* β-actin*: GGC TGTATTCCCCTCCAT CG and CCAGTTGGTAACAATGCCATGT. The relative expression was analyzed with the 2−ΔΔCt method.

### 2.5. Western Blotting

Protein was extracted from cultured podocytes that were treated with RIPA lysis buffer (BestBio, Shanghai, China). The total protein concentration was measured with a BCA protein assay kit (Solarbio, Beijing, China), separated by SDS-PAGE and then transferred to the polyvinylidene difluoride (PVDF) membrane (Milipore, Massachusetts, USA) and blocked with Tris-buffered saline Tween-20 (TBST) containing 5% nonfat milk for 2 h at room temperature. The bands were then incubated with the primary antibodies: antinephrin (1:1000), anti-AKT (1:1000), anti-Phospho-AKT (473) (1:1000), anti-Phospho-AKT (308) (1:1000), anticleaved-caspase3 (1:1000), anti-Nrf2 (1:1000), anti-HO-1 (1:1000), anti-bax (1:5000), anti-bcl-2 (1:2000), antihistone H3 (1:3000), and *β*-actin (1:5000) for 4°C overnight. Next, the bands were incubated with HRP-bounded secondary antibodies (Solarbio, Beijing, China) for 2 h at room temperature and visualized with an ECL detection kit (Biosharp, Shenzhen, China). The *β*-actin was chosen as control.

### 2.6. Immunofluorescence Staining

MPC5 cells were cultured and stimulated in 6-well Chambered Coverglass. After being fixed with 4% paraformaldehyde for 30 min, the MPC5 cells were permeabilized with 0.3% Triton X-100 for 10 min at room temperature and blocked with goat serum for 30 min. Then, the cells were incubated with antiNrf2 (1:200) at 4°C overnight. After being washed three times with PBS, the cells were incubated with FITC-conjugated secondary antibodies (1:200) for 2 h at 37°C. Subsequently, the nucleus was counterstained with DAPI for 10 min at room temperature. The cells were then examined under a fluorescence microscope (Olympus BX63, Japan).

### 2.7. TUNEL Assay

The apoptosis of MPC5 cells was detected by using TUNEL Apoptosis detection kit (Vazyme, Nanjing, China) according to the manufacturer's instructions. TUNEL reaction mixture was added and incubated with cells for 1 h at 37°C. The number of TUNEL-positive nuclei (green) and the total number of nuclei (blue) in each field were scored, and the cells were detected with a fluorescent microscope (Olympus BX63, Japan).

### 2.8. Intracellular ROS Detection

The MPC5 cells were cultured in 6-well Chambered Cover glass and treated as indicated above. The cells were then washed three times with PBS. Next, the cells were incubated with 10 mM fluorescence probe DCHF-DA in PBS at 37°C for 30 min and washed in order to remove the residual probes. The intracellular ROS was detected by a fluorescent microscope (Olympus BX63, Japan).

### 2.9. Mitochondrial ROS Detection

Mitochondrial ROS generation was detected by MitoSOX red (sigma, St. Louis, USA). MPC5 cells were plated in 6-well Chambered Cover glass then were washed with warm Hanks' balanced salt solution three times and incubated in 5*μ*M MitoSOX red for 30min at 37°C. After incubation, the cells were washed twice with Hanks' balanced salt solution. The fluorescent images were examined under a confocal microscope (Leica, Germany).

### 2.10. siRNA Transfection

The siRNA oligo and siRNA negative control (NC) were obtained from GenePharma (Shanghai, China). Transfections were performed with Lipofectamine 2000 reagents (Invitrogen, CA, USA). The concentration of each transfected siRNA was 20 *μ*M, based on the manufacturer's instructions. The culture medium was replaced with serum-free DMEM-F12 medium for 6h, and then it was changed into the original medium.

### 2.11. Statistical Analysis

All data were expressed as means± standard deviations. All analyses were performed using SPSS17.0. For all of the results reported, one-way ANOVAs were conducted followed by Tukey's post hoc test.* P *< 0.05 was considered statistically significant.

## 3. Results

### 3.1. Carnosine Alleviated MPC5 Cell Injury Induced by High Glucose

**Figure 1:**
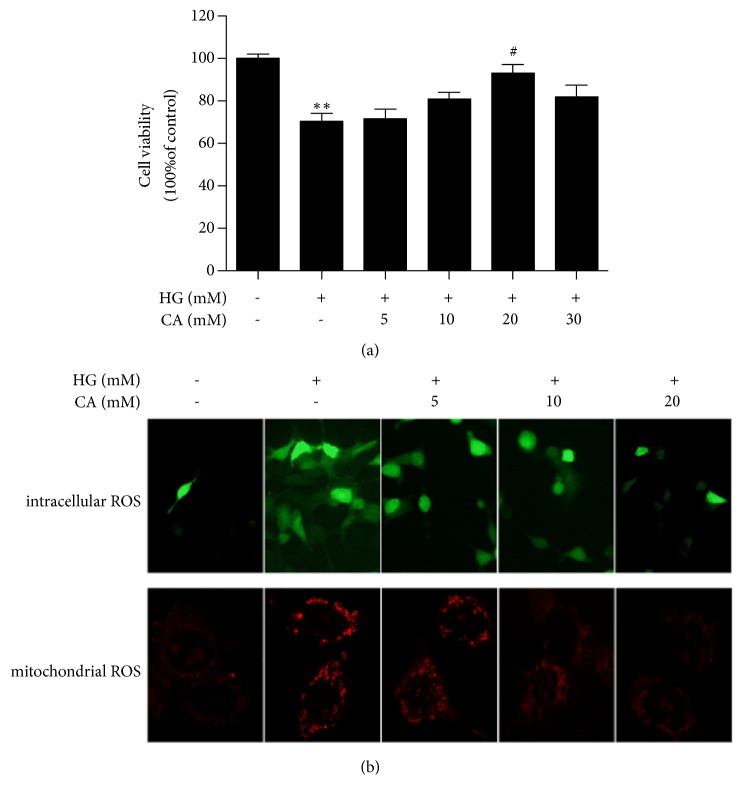
Effects of carnosine on HG-induced cell viability and ROS production in MPC5 cells. Cells were incubated with normal glucose (NG, 5.5 mM), high glucose (HG, 30 mM), and carnosine (5-30mM) under HG condition for 48h. (a) Cell viability was evaluated by CCK-8 assay (n=3). (b) Mitochondrial ROS were detected by the confocal microscope. Intracellular ROS were detected by a fluorescent microscope (n=3). Data are presented as mean ± SD. ^*∗*^*P* < 0.05, ^*∗∗*^*P* < 0.01 vs. NG; ^#^*P* < 0.05, ^##^*P* < 0.01 vs. HG.

As shown in Figure 1(a), results of the CCK8 method revealed that carnosine at concentrations of 5-30mM was incubated on MPC5 cells under HG conditions for 48 hours. The cell viability was ascending in a dose-dependent manner until 30mM. When the HG-incubated podocytes were treated with 20 mM carnosine, the cell viability increased significantly compared with the HG group.

We quantified the intracellular ROS and mitochondrial ROS levels separately, intracellular ROS generation was detected with the fluorescence probe DCHF-DA, and the mitochondrial ROS was examined using a confocal microscope.  Figure 1(b) indicates that the enhanced ROS levels induced by HG were suppressed by carnosine. Therefore, carnosine has strong antioxidant activity to protect MPC5 cell from injury.

### 3.2. Carnosine Inhibited HG-Induced Apoptosis

**Figure 2:**
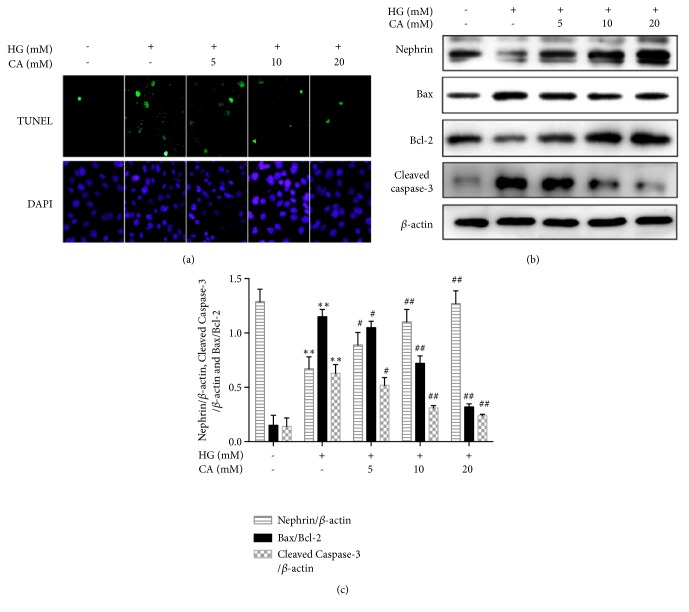
Carnosine protects MPC5 cells from HG-induced apoptosis. (a) Apoptosis of MPC5 cells were detected by TUNEL assay (n=3). Green fluorescence indicates TUNEL-positive and blue indicates DAPI. (b-c) The Western blot showed the protein expression of nephrin, Bax, Bcl-2 and Cleaved caspase-3 in MPC5 cells (n=4). Cells were incubated with normal glucose (NG, 5.5 mM), high glucose (HG, 30 mM), and carnosine (5-20mM) under HG condition for 48h. Data are presented as mean ± SD (n=3). ^*∗*^*P* < 0.05, ^*∗∗*^*P* < 0.01 vs. NG; ^#^*P* < 0.05, ^##^*P* < 0.01 vs. HG.

As shown in Figure 2(a), the apoptosis of MPC5 cells was detected by using TUNEL Apoptosis detection kit. Compared to the normal group, MPC5 cells apoptosis were markedly increased after high glucose incubation for 48h. TUNEL staining also showed that the enhanced apoptosis was significantly suppressed by carnosine in a dose-dependent manner. We also sought to detect whether high glucose induced apoptosis would be associated with mitochondrial apoptotic pathway by Western blotting. Nephrin is an identified protein molecule, which is specifically located on the slit diaphragm. The expression of nephrin is usually used to reflect podocyte cells status. As revealed in Figures 2(b) and 2(c), the expression of nephrin was decreased by high glucose, but it was then enhanced by carnosine. Moreover, the ratio of Bax/Bcl-2 and the expression of Cleaved caspase-3 were significantly decreased in high glucose group plus carnosine.

### 3.3. Carnosine Upregulated PI3K/AKT and Nrf2 Pathways

**Figure 3:**
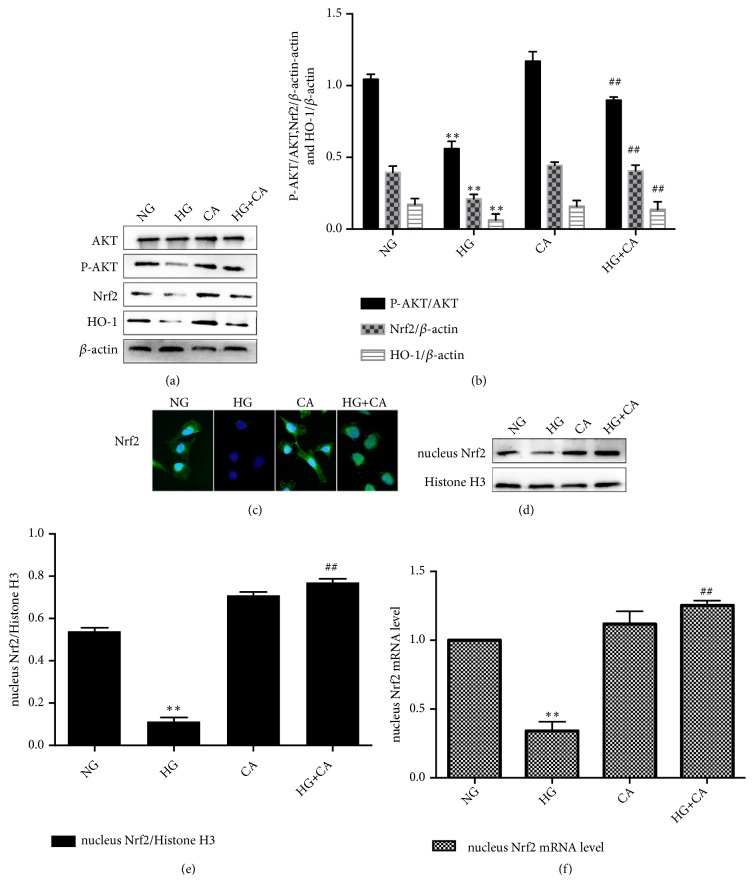
Effects of carnosine on PI3K/AKT and Nrf2 pathways in MPC5 cells. (a-b) The protein expression levels of AKT, P-AKT, Nrf2, and HO-1 were detected by Western blot (n=3). (c) The effects of carnosine on the expression of Nrf2 in MPC5 cells were detected by immunofluorescence (n=3). (d-f) The protein expression of Nrf2 in nucleus was detected by Western blot (n=3); the mRNA expression of Nrf2 in nucleus was analyzed by RT-qPCR (n=3). NG: normal glucose 5.5mM; HG: high glucose 30mM; CA: carnosine 20mM; HG+CA: high glucose (30mM) plus carnosine (20mM). Data are presented as mean ± SD. ^*∗*^*P* < 0.05, ^*∗∗*^*P* < 0.01 vs. NG, ^#^*P* < 0.05, ^##^*P* < 0.01 vs. HG.

PI3K/AKT and Nrf2 pathways have been found to play a pivotal role in the antiapoptosis [17]. To further verify the effect of carnosine on PI3K/AKT and Nrf2 pathways. MPC5 cells were divided into four groups with different treatments: normal glucose (NG, 5.5 mM), high glucose (HG, 30 mM), carnosine (CA, 20mM), and HG plus carnosine (CA, 20mM). We examined the protein expression levels of AKT, p-AKT, Nrf2, and HO-1. Compared with the NG group, the expression of Nrf2, HO-1, and p-AKT protein was significantly decreased in HG group, but these were lately upregulated by carnosine treatment (see Figures 3(a) and 3(b)). To determine whether carnosine would affect the nuclear translocation of Nrf2, we performed cellular immunofluorescence assay. Nrf2 was predominantly located in the cytoplasm of MPC5 cells in the NG group. As shown in Figure 3(c), the fluorescence intensity of the nuclear Nrf2 was greatly descended in HG group, whereas elevated in HG+CA group. The protein expression of nuclear Nrf2 was significantly enhanced in HG+CA group compared with HG group. RT-qPCR results were consistent with the results of Western blot (Figures 3(d)–3(f)). The results revealed that carnosine could upregulate PI3K/AKT and Nrf2 pathways under HG condition.

### 3.4. Nrf2 Pathway Inhibited by PI3K/AKT to Attenuate MPC5 Cell Injury of Carnosine

**Figure 4:**
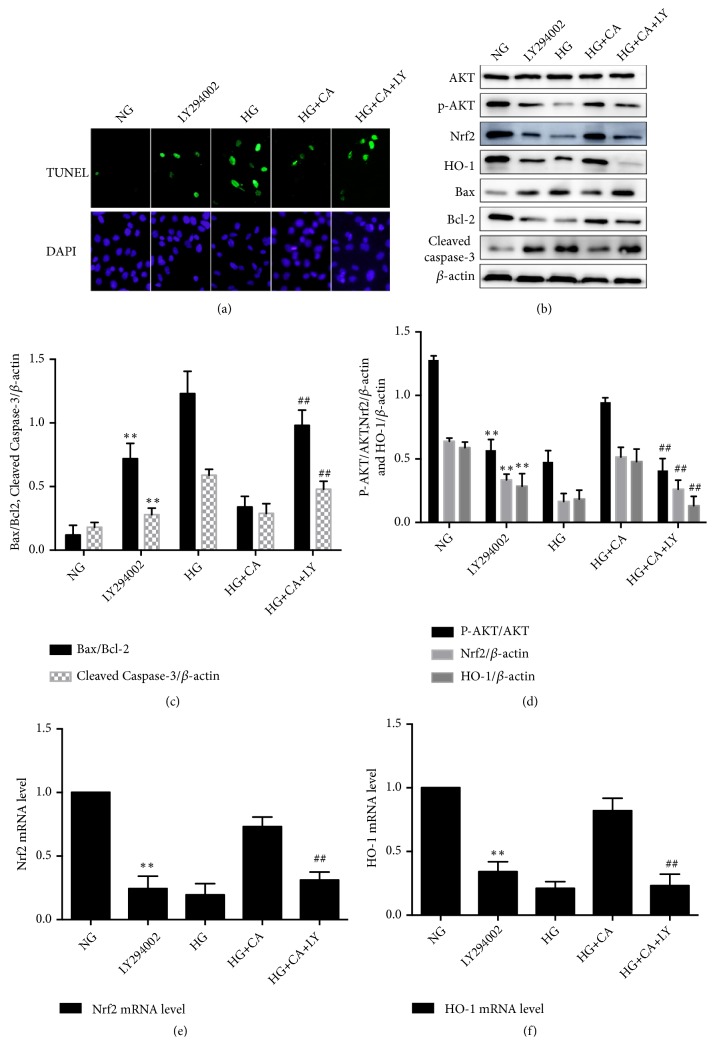
Effect of PI3K inhibitor on carnosine effect against HG-induced apoptosis. (a) Apoptosis of MPC5 cells was detected by TUNEL assay (n=3). (b-d) The protein expression levels of AKT, P-AKT, Nrf2, HO-1, Bax, Bcl-2, and Cleaved caspase3 were detected by Western blot (n=3). (f) The mRNA expression of Nrf2 and HO-1 analyzed by RT-qPCR (n=3). NG: normal glucose 5.5mM; LY294002: 20*μ*M; HG: high glucose 30mM; HG+CA: high glucose (30mM) plus carnosine (20mM); HG+CA+LY: high glucose (30mM) plus carnosine 20mM) plus LY294002 (20*μ*M). Data are presented as mean ± SD. ^*∗*^*P* < 0.05, ^*∗∗*^*P* < 0.01 vs. NG; ^#^*P* < 0.05, ^##^*P* < 0.01 vs. HG+CA.

To further investigate whether the PI3K/AKT and Nrf2 pathways are associated with carnosine's protective effects, the cells were pretreated with LY294002 (20*μ*M), a specific inhibitor of PI3K/AKT pathway. MPC5 cells were divided into five groups with different treatments: NG, LY294002, HG, HG plus carnosine (CA, 20mM), HG plus carnosine (20mM) plus LY294002 (20*μ*M).  Figure 4(a) show that apoptosis cells as assessed by TUNEL staining were significantly more elevated in the LY294002 group than in the NG group. LY294002 may depress the protective effect of carnosine on HG-induced apoptosis. Figures 4(b)–4(d) showed that the protein expression levels of Nrf2, HO-1, AKT, P-AKT, Bax, Bcl-2 and Cleaved caspase-3. LY294002 enhanced the expression of Cleaved caspase-3 protein and descended the expression of Nrf2, HO-1 protein. The P-AKT/AKT ratio was markedly decreased in MPC5 cells exposed to LY294002. The Bax/Bcl-2 ratio was significantly increased in the LY294002 group and the HG plus carnosine plus LY294002 group, respectively. The RT-qPCR results, shown in Figures 4(e) and 4(f), demonstrated that Nrf2 and HO-1 mRNA levels were indeed induced by LY294002 treatment and were associated with the alternations of protein levels. In light of the above findings, we concluded that LY294002 could inhibit Nrf2 signaling pathway by inhibiting AKT phosphorylation. Carnosine protected MPC5 cell against HG-induced apoptosis mainly through PI3K/AKT and Nrf2 signaling pathways.

### 3.5. Knockdown Nrf2 or Inhibiting PI3K/AKT Attenuated the MPC5 Cell Protective Effect of Carnosine.

**Figure 5:**
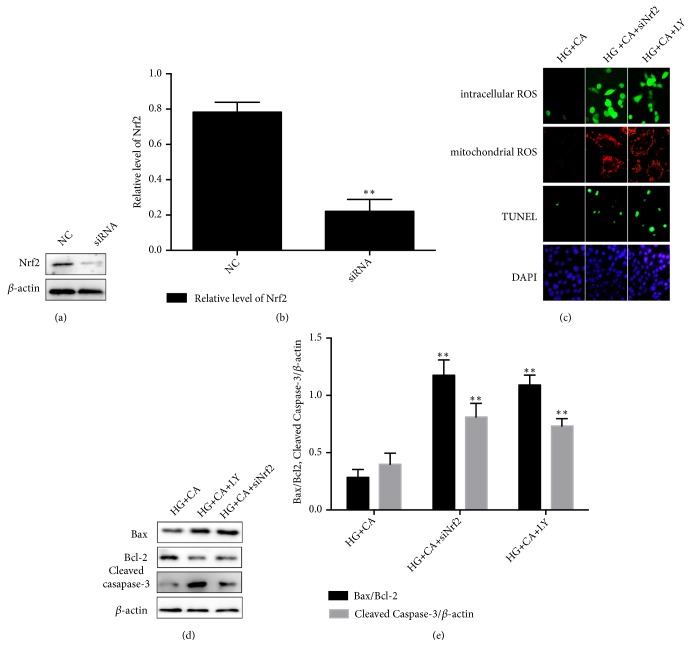
Effect of siNrf2 and PI3K inhibitor on carnosine effect against HG-induced ROS production and cell apoptosis. (a-b) Western blots were performed to detect siNrf2 expression in MPC5 cells (n=3). (c) Mitochondrial ROS were detected by the confocal microscope (n=3). Intracellular ROS were detected by a fluorescent microscope (n=3). Apoptosis of MPC5 cells was detected by TUNEL assay (n=3). (d-e) The protein expression levels of Bax.Bcl-2 and Cleaved caspase-3 were detected by Western blot (n=3); NC: siRNA negative control; HG+CA: high glucose (30mM) plus carnosine (20mM); HG+CA+siNrf2: high glucose (30mM) plus carnosine (20mM) plus siNrf2 (20*μ*M); HG+CA+LY: high glucose (30mM) plus carnosine (20mM) plus LY294002 (20*μ*M); data are presented as mean ± SD. ^*∗*^*P* < 0.05, ^*∗∗*^*P* < 0.01 vs. HG+CA.

To determine the antioxidant and antiapoptosis effects of Nrf2 and PI3K/AKT on MPC5 cells exposed to carnosine with HG environment, we transfected siNrf2 into podocytes. The Western blot detected the protein expression of siNrf2 that was significantly decreased compared with NC group, indicating the success of Nrf2 knockdown (see Figures 5(a) and 5(b)). MPC5 cells were divided into three groups with different treatment: HG plus carnosine (CA, 20mM), HG plus carnosine (20mM) plus siNrf2 (20*μ*M), and HG plus carnosine (20mM) plus LY294002 (20*μ*M). The levels of ROS and the apoptotic cells in siNrf2 and LY294002 group were higher than those in carnosine group, which suggested that Nrf2 and PI3K/AKT were important antioxidant targets of carnosine (Figure 5(c)).

Furthermore, we observed the expression levels of the markers associated with apoptosis, as shown in Figures 5(d) and 5(e). Although there was no significant difference between siNrf2 and LY294002 group, the ratio of Bax/Bcl-2 and the expression of Cleaved caspase-3 were significantly increased in both siNrf2 and LY294002 groups compared to the carnosine group. Correspondingly, the TUNEL stain indicated that the HG-induced apoptosis cells were enhanced in siNrf2 and LY294002 conditions, compared with only carnosine treatment. Taken together, it can be concluded that both PI3K/AKT and Nrf2 play a mediating role through which effects of carnosine protect MPC5 cells against HG-induced ROS and cell apoptosis.

## 4. Discussion

Podocytes are epithelial cells of the glomerular visceral layer that adhere to the surface of the glomerular basal layer and are terminally differentiated cells [18]. In recent years, podocyte injury was found to be involved in the occurrence and development of diabetic nephropathy, mainly manifested as podocyte exfoliation, apoptosis, and foot process fusion [19]. Oxidative stress is involved in the pathophysiology of diabetic nephropathy. Brownlee et al. [20] proposed a unifying mechanism for the pathobiology of diabetic complications, such that excessive ROS produced by mitochondrial damage could activate polyol pathway, advanced glycation end products pathway, protein kinase C pathway and hexamine metabolic pathway, all of which may lead to hyperglycemia-induced vascular and kidney damage.

Mitochondria increases ROS production under high glucose. The increased ROS oxidizes and modifies mitochondrial membrane and related proteins, and as a result membrane potential is reduced and membrane permeability is increased, respectively. The above processes, in turn, lead to a leakage of apoptotic effectors such as cytochrome C from mitochondria. Finally, both procaspase 9 and procaspase 3 are activated, indicating podocyte apoptosis.

Carnosine has antioxidant activity, and its protective effect on diseases has been extensively studied. Carnosine treatment has been found to decrease high malondialdehyde and diene conjugate and protein carbonyl levels and lead to significant increases in vitamin E level and SOD activity in the liver of 22 months old rats [21]. Fouad et al. [22] demonstrated that carnosine could significantly attenuate malondialdehyde and glutathione in cisplatin-induced acute renal damaged mice, with histopathological examination and scoring indicating that carnosine markedly ameliorated cisplatin-induced renal tubular necrosis.

In the current study, we examined the effects of carnosine on HG-induced oxidative stress and cell apoptosis* in vitro*. Moreover, our study is the first to demonstrate the protective effects of carnosine HG-induced podocyte injury through both PI3K/AKT and Nrf2 pathways. We chose the concentration range of 5-30mM carnosine under HG condition for cell viability. Carnosine at the concentrations of 20 mM significantly increased the cell viability compared with the HG group. The decrease of cell viability by 30 mM carnosine may be related to its inhibition of cell cycle and reduction of DNA synthesis [23]. That is, in podocytes there were significant increases of ROS production under HG condition. However, carnosine decreased the ROS production in HG-induced podocytes in a dose-dependent manner. The increased production of ROS induced by HG was reduced by carnosine treatment (at 20mM). Therefore, in this study 20mM was the chosen concentration for subsequent assays. In the current study, the cell apoptosis was detected by TUNEL assay; TUNEL stain showed that HG-induced apoptosis positive cells increased dramatically than that of NG. Carnosine decreased HG-induced apoptosis positive cells in a dose-dependent manner. The expression of Cleaved caspase-3 is usually used to reflect apoptosis status [24]. The expression of Cleaved caspase-3 was enhanced in HG group than NG group, but it was then attenuated by carnosine.

Nephrin is a biomarker of podocytes which is closely related to the maintenance of glomerular filtration barrier [25]. Yang et al. [26] detected the role of miR-218 in podocyte apoptosis that the expression of nephrin was downregulated in the HG-treated podocytes compared to the NG-treated podocytes. Our findings reveal that carnosine could increase the expression of nephrin to against HG-induced podocytes cell injury.

Nrf2 is a master regulator of antioxidative responses [27], which participates in the pathophysiological process of oxidative stress in many diseases [28, 29]. PI3K/AKT signaling pathway can also regulate the activity of Nrf2. Phosphorylation of AKT can enhance the activity of downstream Nrf2, thereby promoting endogenous antioxidant activity of cells [30]. The current findings revealed that carnosine could reverse the decreased expression of Nrf2 in HG condition which also upregulates the PI3K/AKT. LY294002, a specific AKT inhibitor, downregulates the P-AKT, Nrf2, and HO-1, by which the protective effect of carnosine against HG-induced apoptosis is blocked. Therefore, knockdown Nrf2 or inhibiting PI3K/AKT attenuates the antioxidant and antiapoptosis effect of carnosine. In other words, the protective effect of carnosine might be attributable to its antioxidant properties through upregulating the activity of AKT and activating the Nrf2/HO-1 pathway.

In conclusion, the current findings demonstrate that carnosine protects MPC5 cell against HG-induced apoptosis through inhibiting ROS generation by activation of PI3K/AKT and Nrf2 pathways. It reminds us this finding may be involved in carnosine therapeutic mechanisms in diabetic nephropathy.

## Data Availability

The data used to support the findings of this study are available from the corresponding author upon request.
